# Liquid Crystal Elastomer for Compression Therapy

**DOI:** 10.1002/adhm.202402881

**Published:** 2024-12-04

**Authors:** Gaoweiang Dong, Fangchen Zhao, Zongyu Gao, Shengqiang Cai

**Affiliations:** ^1^ Materials Science and Engineering Program University of California La Jolla San Diego CA 92093 USA; ^2^ Department of Mechanical and Aerospace Engineering University of California La Jolla San Diego CA 92093 USA

**Keywords:** compression therapy, dynamic stocking, liquid crystal elastomer, static stocking

## Abstract

Compression therapy is a widely used treatment for various disorders including venous leg ulcers. Traditional methods such as inelastic bandages and elastic stockings, have limitations in maintaining optimal pressure over time. Dynamic therapy devices offer intermittent pressure cycles but are often bulky or rigid. Here liquid crystal elastomer (LCE) is proposed for both static and dynamic compression therapy. Due to the soft elasticity of polydomain LCE, LCE‐based static stocking can maintain consistent pressure over a wide range of leg diameters, permitting the tolerance of stocking application inconsistencies, various limb sizes, and interfacial pressure drop due to leg deswelling. The LCE‐based dynamic stocking consists of monodomain LCEs with reversible thermal actuation, heating elements, and electronics. The dynamic stocking generates intermittent pressure from 20 to 60 mmHg with a slight temperature increase above 33 °C and offers pressure profile programmability. Furthermore, an untethered LCE‐based dynamic compression device on a human leg is demonstrated. Both LCE‐based static and dynamic stockings show minimal stress relaxation and reusability over 1000 cycles, ensuring long‐term use in compression therapy applications.

## Graphical abstract

This study introduces liquid crystal elastomer (LCE) for both static and dynamic compression therapy. The soft elasticity of polydomain LCE permits the LCE‐based static stocking consistent pressure as the limb size changes. The thermal actuation of monodomain LCE allows the LCE‐based dynamic stocking to generate controllable pressure cycles. An untethered LCE‐based dynamic compression device is demonstrated on a human leg.

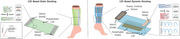

## 1 Introduction

Compression therapy, a widely used treatment method, involves applying consistent pressure to the limbs or other body parts using specially designed garments, bandages, or devices. The applied pressure facilitates better blood flow and lymphatic drainage, supporting the natural healing processes in the body.^[^ ^1^ ^]^ The therapy is commonly used for managing a wide range of disorders, including chronic venous diseases, edema, and conditions associated with poor blood circulation. The key to effective compression therapy is applying the appropriate level of pressure for a specified duration.^[^ ^2^ ^]^ Inadequate pressure can delay treatment, while excessive pressure may cause pain or tissue damage.^[^ ^3^ ^]^

Traditional compression therapies primarily utilize either inelastic bandages or elastic stockings. While inelastic compression bandaging offers low cost and versatile application, it relies heavily on the stocking application skills of the nurse and may lose elasticity over time.^[^ ^4^, ^5^ ^]^ For instance, Keller et al.^[^ ^6^ ^]^ carried out research involving 21 nurses to assess their precision in applying inelastic compression bandages. The findings indicated that 34.9% of the applications resulted in inadequate sub‐bandage pressure levels when the application was only based on the experience of the nurses. Elastic stockings, easier to apply and more patient‐friendly, depend significantly on limb size for effective pressure application. Moreover, as the swelling in the treated limb decreases, the limb's diameter reduces and consequently, both inelastic bandages and elastic stockings, experience a reduction in applied pressure as they conform to the smaller limb size.^[^ ^7^ ^]^ For example, Mosti et al.^[^ ^8^ ^]^ conducted a study on pressure reduction during deswelling in 30 patients with chronic leg edema. The findings revealed that the minimum pressure drop observed was 21%, resulting from a 10% reduction in calf circumference. Such pressure drop is undesired since a proper level of compression over the wounded area ensures more effective compression treatment for venous leg ulcers and oedema reduction.^[^ ^9^ ^]^

To enhance pressure control and compliance with the bandage/stocking, researchers have explored dynamic compression therapy devices incorporating an actuating component. Moreover, by adopting actuators, dynamic compression therapy can be realized with intermittent pressure cycles, which was shown to be more effective than static compression.^[^ ^10^, ^11^ ^]^ Pneumatic compression has been extensively studied as a dynamic compression therapy device, capable of delivering precise and dynamic pressure to the limb. For instance, Hakala et al.^[^ ^12^ ^]^ employed adjustable air bladders in their design of a compression stocking, enabling fast pressure change within a few seconds. However, these devices are bulky, noisy, and usually not portable. Dynamic compression therapy devices utilizing motor‐driven mechanisms can produce substantial torque and demonstrate rapid actuation speeds. For instance, Rahimi et al.^[^ ^13^ ^]^ created a motorized compression bandage that incorporates tensioning wires capable of exerting pressures up to 87.3 mmHg. Nonetheless, integrating the motor into a compression garment poses challenges, particularly when applying high torque.^[^ ^14^ ^]^ Shape memory alloys (SMA) provide the advantage of seamless textile integration and a low‐profile design. For example, Duvall et al.^[^ ^15^ ^]^ developed a dynamic compression vest that employs shape memory alloy, achieving compression pressure up to 52.5 mmHg. However, the operational temperature of SMA typically exceeds human body temperature by tens of degrees, potentially posing risks during extended use. Additionally, motor‐driven‐based and SMA‐based compression therapy devices mentioned are inherently rigid actuators, which reduce their compliance and comfort as wearable devices for humans.

Recently, soft actuating polymers have also been explored in constructing dynamic compression therapy devices. Pourazadi et al.^[^ ^16^ ^]^ utilized dielectric elastomeric actuators (DEA) to develop a dynamic compression bandage that allows for precise pressure control. However, the pressure variation of the device is limited to 10 mmHg, and it operates at a high voltage exceeding 2 kV, raising safety concerns. Ross et al.^[^ ^17^ ^]^ developed a compression device based on liquid crystal elastomer, consisting of a microprocessor, an active layer, a stimuli layer, and an insulation layer. However, the working temperature of the liquid crystal elastomer exceeds human body temperature by over 30 °C, and the fabrication and performance of the device have not been carefully studied. Shape memory polymers (SMP) have recently emerged as promising materials for smart compression therapy due to their significant actuation stress and inherent softness. Kumar et al.^[^ ^18^ ^]^ and Ahmad et al.^[^ ^19^ ^]^ developed an SMP‐based compression therapy device and demonstrated ≈a 10 mmHg increase of interfacial pressure with increasing temperature. However, these devices exhibited notable stress relaxation, exceeding 40%, which could undermine their therapeutic efficacy. To address the issue of stress relaxation, Kumar et al.^[^ ^20^ ^]^ and Narayana et al.^[^ ^21^, ^22^ ^]^ implemented a preconditioning step in their SMP‐based devices, significantly reducing stress relaxation and ensuring minimal pressure decay during dynamic compression treatment. Despite this advancement, each intermittent pressure cycle exceeds half an hour and the required preconditioning time of at least one hour before application poses practical challenges, limiting the immediate usability and convenience of SMP‐based compression therapies. More importantly, most shape memory polymers turn to glassy states at room temperature, which makes the SMP‐based dynamic stocking stiff and difficult to wear.

Herein, we introduce room‐temperature responsive liquid crystal elastomer (LCE) as a material for both static and dynamic compression therapy applications. LCE is an elastomer, composed of liquid crystal mesogen, chain extender, and crosslinker, exhibiting unique thermomechanical properties that differentiate it from traditional elastomers.^[^ ^23^ ^]^ LCEs can be synthesized in two different states at room temperature: a polydomain with randomly oriented mesogen domains, and a monodomain with aligned mesogen domains.

For static compression therapy, we propose the polydomain LCE as the elastic material due to its unique soft elasticity, caused by the rotation of liquid crystal mesognes.^[^ ^24^ ^]^ It demonstrates a stress–strain curve with a stress plateau and negligible hysteresis in loading and unloading cycles. Consequently, a static stocking made from a polydomain LCE can maintain a constant pressure within a large range of stretch, offering a significant tolerant range for application inconsistencies compared to inelastic bandages and accommodating a variety of limb sizes compared to elastic stockings. Moreover, the negligible hysteresis of the polydomain LCE during loading and unloading allows the LCE‐based static stocking to maintain a steady pressure as the limb circumference decreases due to the deswelling. Additionally, the negligible stress relaxation and good cyclability of the polydomain LCE permit the maintenance of the pressure level without requiring external energy or additional tightening steps, which is a notable advantage over other compression devices. We created a table comparing existing static compression therapy solutions discussed above with our proposed LCE‐based static stocking in Table S1 (Supporting Information).

For dynamic compression therapy, the monodomain LCE presents promising characteristics due to its reversible thermal actuation properties. Upon heating to slightly above human body temperature, a monodomain LCE undergoes the nematic‐isotropic phase transition, enabling it to exert substantial actuation stress when the displacement is fixed. By incorporating a power supply, a sensing unit, a controller, and a stretchable heater into the monodomain LCE‐based stocking, it can achieve intermittent compression cycles, enhancing therapeutic efficacy. More importantly, the monodomain LCE maintains its actuation properties >1000 cycles, exhibiting minimal stress relaxation and superior durability compared to traditional shape memory polymers. We have also created a table comparing all the dynamic compression therapy solutions discussed above with our proposed LCE‐based dynamic stocking in Table S2 (Supporting Information).

## 2 Results and Discussion

### 2.1 Design Concept

**Figure 1:**
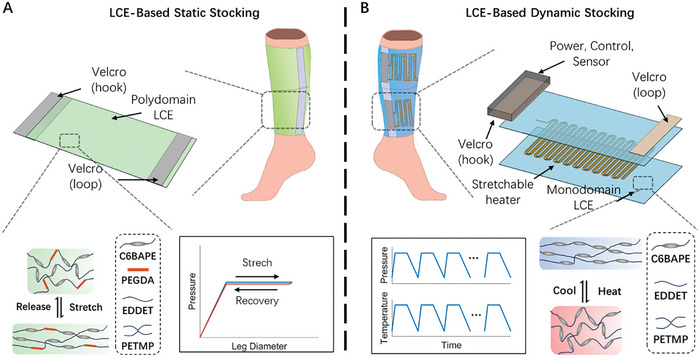
Design concept and working mechanisms of LCE‐based static and dynamic compression stockings. A) An LCE‐based static stocking consists of polydomain LCEs and Velcro strips. The pressure remains nearly constant over a wide range of leg diameters, corresponding to the liquid crystal mesogen rotation in the elastomer, as shown in the molecular schematics. B) An LCE‐based dynamic compression stocking consists of monodomain LCEs, a stretchable heater, a Power, Control, Sensor (PCS) module, and Velcro strips. With periodic voltage input, the LCE‐based dynamic stocking can generate a cyclic, consistent, and controlled pressure profile within the human‐comfort temperature range. The pressure increase is due to the nematic‐isotropic phase transition of the monodomain LCE, as shown in the molecular schematics.

As shown in **Figure** **1**A, we employed a polydomain LCE for static compression therapy, distinguishing itself from previously used materials, including inelastic bandages and elastic stockings. First, when a polydomain LCE is subjected to uniaxial stretch, its stress initially increases with strain, followed by a stress plateau over a wide range of strain. This property enables the LCE‐based static stocking to maintain a constant pressure level (Figure 1A), demonstrating a high tolerance for application inconsistencies compared to traditional inelastic bandages. Moreover, an LCE‐based static stockings can easily accommodate various limb sizes while providing similar interfacial pressure. This contrasts with the conventional solutions where the stress in the material increases with strain monotonically so that the applied pressure varies a lot for different limb sizes.

Second, the polydomain LCE with a carefully tailored composition shows negligible stress reduction during unloading due to the extremely small mechanical hysteresis compared to conventional stocking materials (Figure 1A). Such features of the polydomain LCE can be important as a swollen leg starts to deswell after the application of the compression stocking. When traditional compression materials are used, a decrease in limb size often leads to a significant drop in applied pressure, which may diminish the effectiveness of treatment.^[^ ^8^ ^]^ In contrast, a polydomain LCE can maintain a stable pressure level during the reduction in limb size. The ability to maintain constant pressure during both application and size reduction allows the LCE‐based static stocking to provide adequate compression across various limb sizes, providing a new solution compared to conventional materials.

Finally, the polydomain LCE demonstrates additional characteristics that make it suitable for static compression therapy. Notably, it maintains its stress level throughout the duration of its application, which typically spans a few h. Furthermore, the polydomain LCE exhibits a consistent stress profile across hundreds of usage cycles.

As illustrated in Figure 1B, the monodomain LCE has merits in dynamic compression therapy due to its thermomechanical properties. We constructed an LCE‐based dynamic stocking using a monodomain LCE and a heating element. We further developed a Power, Control, Sensor (PCS) module to control the LCE‐based dynamic stocking and collect real‐time data on pressure, temperature, and voltage. The monodomain LCE exhibits reversible actuation stress in response to temperature changes across the nematic‐isotropic transition temperature (T_NI_). By adjusting the chemical composition of the LCE, we tuned the T_NI_ to slightly above human body temperature, allowing the LCE‐based dynamic stocking to generate sufficient interfacial pressure for therapeutic purposes^[^ ^2^ ^]^ upon a mild increase in temperature. We fabricated the heating element from a thin copper film and precisely shaped it using a contour cutter to ensure that it provides a uniform temperature distribution. The heater can withstand moderate stretching and compression, maintaining consistent performance through thousands of operational cycles without noticeable changes in its electrical resistance. The application of periodic voltage enables the stocking to generate compression cycles consistently, maintaining adequate pressure without decay over time. Additionally, we employed pressure feedback mechanisms to regulate the interfacial pressure profile, permitting the controllability of the pressure profile throughout each compression cycle.

### 2.2 The LCE‐Based Static Stocking

#### 2.2.1 Stress–Strain Relationship of a Polydomain LCE

We first examined the mechanical properties of a polydomain LCE at 33 °C, a temperature close to human skin temperature. The mechanical characterization of a polydomain LCE under this temperature can provide the necessary guidance for the design of static stockings. To identify the chemical composition of an LCE that exhibits a stress plateau and minimal hysteresis, we substituted a portion of rigid liquid crystal mesogen C6BAPE with a more flexible molecule, PEGDA.

**Figure 2:**
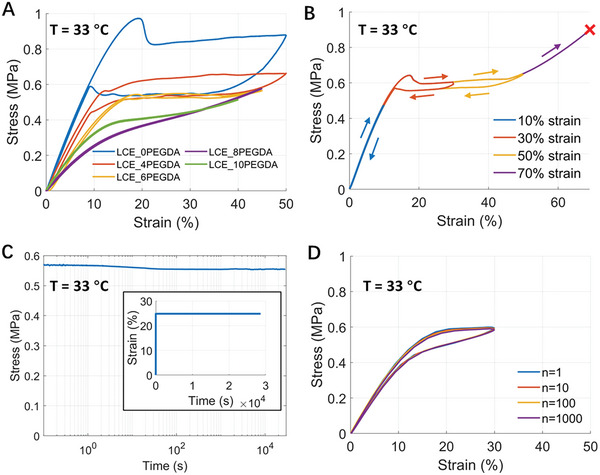
Stress–strain characterization of a polydomain LCE at 33 °C. A) Loading and unloading of a polydomain LCE with various PEGDA content. B) Loading and unloading of LCE_6PEGDA polydomain LCE at various strain values where LCE breaks during the 70% strain loading. C) Stress relaxation of LCE_6PEGDA polydomain LCE under 30% applied strain. D) Durability characterization of LCE_6PEGDA polydomain LCE.

As shown in **Figure** **2**A, the loading and unloading of various polydomain LCEs at 33 °C illustrate the effect of increasing PEGDA content on the hysteresis loop and plateau behavior. Specifically, the number in the sample name represents the percentage of the PEGDA acrylate group in the elastomer. For example, the “LCE_4PEGDA” indicates that the PEGDA contributes 4% of the total acrylate groups among all the chemicals. The detailed chemical composition of each sample can be found in Text S1 (Supporting Information). As the PEGDA content increases from the LCE_0PEGDA to the LCE_10EPGDA sample, we can observe the hysteresis and plateau stress decrease and eventually disappear for the LCE_6PEGDA sample. The small hysteresis observed in LCE_6PEGDA sample results from the combination of the appropriate amount of crosslinking density, liquid crystal mesogens, and PEGDA molecules used in synthesizing the LCEs. Compared to previous studies, the LCE we synthesized in the current work has much higher crosslinking density, which contributes to its low mechanical hysteresis. The increase of PEGDA content also affects the T_NI_ of the LCE, which is shown in differential scanning calorimetry tests in Figure S1 (Supporting Information). As a result, we selected an LCE_6PEGDA for the LCE‐based static stocking due to its demonstrated stress plateau and reduced hysteresis loop, which are advantageous for accommodating application inconsistencies, fitting various limb sizes, and mitigating pressure reduction associated with limb deswelling.

As shown in Figure 2B, we conducted loading and unloading tensile tests of the LCE_6PEGDA sample at various strain levels. During the loading of the LCE sample, the stress first increases with strain and then reaches a stress plateau ≈0.6 MPa, followed by a further increase in stress and eventually fails. The polydomain LCE exhibits elastic deformation, which is usually desired for static compression stocking.^[^ ^25^ ^]^ Figure 2C presents the stress relaxation behavior of a polydomain LCE when subjected to a 25% strain and maintained for ≈8 h, showing minimal stress relaxation and indicating the ability of the material to sustain pressure within the stocking. As shown in Figure 2D, we further conducted durability assessments through cyclic loading and unloading tests for 1000 cycles. To save the experimental time, we adopted a strain rate of 10% s^−1^ for the tests. The results indicate a decay in the magnitude of the plateau stress after 1000 cycles of <2%. The good durability of the polydomain LCE is comparable to other elastic components in common elastic knitted fabrics, such as spandex and elastane,^[^ ^26^ ^]^ highlighting its potential for long‐term use in compression therapy applications.

#### 2.2.2 Performance of an LCE‐Based Static Stocking

The chemical composition of the LCE can be tailored to exhibit particular mechanical properties so that it can meet specific interfacial pressure requirements for compression stocking applications. Based on the force balance, the hoop stress^[^ ^27^ ^]^ defines the interfacial pressure generated by the LCE stocking as:

(1) $$P=\frac{2st}{D}$$

where *P* is the interfacial pressure applied by the stocking, *t* represents the thickness of the LCE layer, *D* is the diameter of the limb or stocking, and *s* is the hoop stress in the LCE, which is a function of *D*. By adjusting the chemical composition of the LCE, it is possible to modulate *s* under various working temperatures, consequently, tailor *P* to meet the desired compression therapeutic objectives. The ability to fine‐tune the thermomechanical properties of an LCE underscores the adaptability of the material and its potential utility in creating effective compression therapies, both static and dynamic.

**Figure 3:**
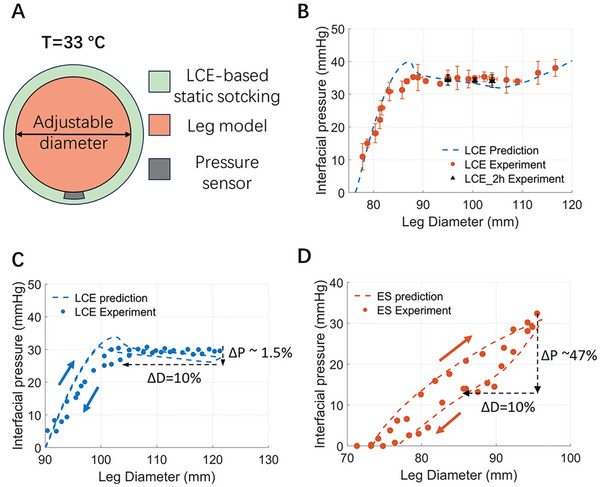
Performance of an LCE‐based static stocking. A) Schematics of the measurement of interfacial pressure generated by the LCE‐based static stocking. B) Prediction and measurement of interfacial pressure over a wide range of leg diameters of LCE‐based static stocking. Prediction and measurement of interfacial pressure drop during the unloading of C) an LCE‐based static stocking and D) a representative commercial elastic stocking.

With the understanding of the stress plateau and small hysteresis of the polydomain LCE at human skin temperature, we characterized the interfacial pressure of the LCE‐based static stocking as a function of leg diameter. **Figure** **3**A illustrates the experimental setup, where a leg model with adjustable diameters was utilized, by using rubber bands to secure the removable rods and ensure a consistent circular cross‐sectional profile. We took the interfacial pressure measurements using a 1mm‐thick homemade pressure sensing pouch placed between the LCE and the leg model, guaranteeing minimal distortion of the stocking's curvature. Figure S2 (Supporting Information) illustrates experimental pictures of pressure measurements.

To understand the performance of the LCE‐based static stocking, we predicted the interfacial pressure using Equation (1), where the stress *s* in an LCE is a function of the circumference of an LCE stocking π*D*, where *D* is the leg diameter. Compression therapy requires 30 to 40 mmHg of interfacial pressure to have a therapeutic effect.^[^ ^28^ ^]^ Therefore, we chose 30 mmHg as the target interfacial pressure level for the study. By using Equation (1) and the LCE stress plateau magnitude *s*, we can calculate the thickness of the LCE stocking and fabricate it accordingly. Additionally, we chose a representative leg diameter of 105 mm for the study of an LCE‐based stocking since it is close to the average calf size for humans.^[^ ^29^ ^]^ To generate consistent interfacial pressure levels within a wide range of leg diameter change, the polydomain LCE strain should fall within the corresponding strain range for the stress plateau, which is 20% to 50% according to Figure 2B. Therefore, the initial diameter and thickness of the LCE‐based static stocking can be determined.

Figure 3B illustrates the comparison between predicted and measured interfacial pressures generated by the LCE stocking on the leg model with varying diameters. We used an LCE stocking with a length of 234.2 mm and a thickness of 0.36 mm in the prediction and experiment. Both the predicted and measured interfacial pressure are approximately constant for the leg diameter ranging from 89 to 115 mm, a span sufficient to cover typical inconsistencies in the bandage application and the size range of commercial elastic stockings. We have also measured the consistency of the generated interfacial pressure over two hours for the leg model with diameters of 95, 100.5, and 104 mm. Our measurements show minimal pressure reduction, which implies the great stability of the LCE‐based static stocking.

As shown in Figure 3C, to characterize the pressure drop during the leg deswelling, we predicted and measured the interfacial pressure generated by the LCE stocking with first increase and then decrease of the diameter of the leg model. The LCE‐based static stocking exhibited only a 1.5% pressure drop with a 10% reduction in diameter. We further conducted similar experiments and predicted the interfacial pressure for a representative commercial elastic stocking (ES in Figure 3D). To predict the pressure as a function of leg diameter for ES, we conducted a tensile test on the calf area of ES along the course direction. The tensile test results of ES can be found in Figure S3 (Supporting Information). As illustrated in Figure 3D, in contrast to the LCE stocking, the commercial elastic stocking showed a 47% pressure drop under the same experimental conditions, which is significantly larger than that of the LCE‐based static stocking. The commercial stocking was purchased from Amazon Basic Care in medium size (Amazon.com Services LLC.). The error bars of Figure 3C,D are detailed in Figure S4 (Supporting Information). Therefore, an LCE‐based static stocking is a good candidate for maintaining the pressure level during leg deswelling compared to commercial elastic stockings.

### 2.3 An LCE‐Based Dynamic Stocking

#### 2.3.1 Actuation Stress of a Constrained Monodomain LCE

We selected the monodomain LCE as the actuating material to construct the LCE‐based dynamic stocking. In contrast to the polydomain LCE, we did not include PEGDA in the polymer network so that we could fully take advantage of the phase transition of liquid crystal mesogen to generate a large actuation force.^[^ ^30^ ^]^ To understand the actuating behavior of a monodomain LCE, we conducted a series of thermomechanical characterizations.

**Figure 4:**
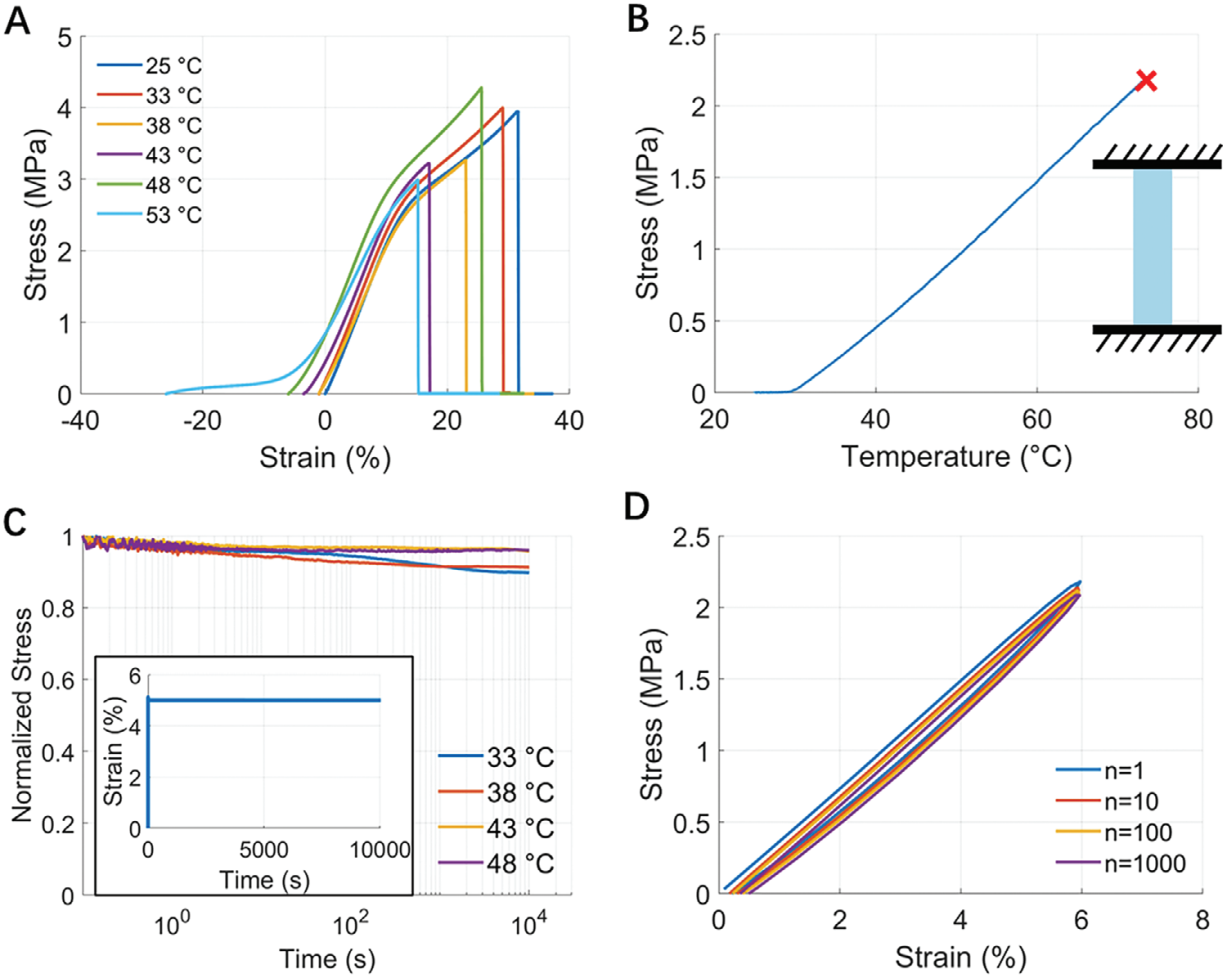
Thermomechanical characterization of a monodomain LCE. A) Stress–strain relationships of monodomain LCEs at various temperatures. The length of a freestanding LCE sample at 25 °C is used as the reference to calculate the strain. B) The thermal actuation stress of a monodomain LCE increases with increasing temperature when the length is fixed at the initial state. C)Stress relaxation of a monodomain LCE with 5% strain under various temperatures. D) Loading and unloading stress–strain curves of a monodomain LCE after different numbers of loading cycles.

**Figure** **4**A presents the thermomechanical properties of a monodomain LCE, depicting its response to uniaxial tensile tests across various temperatures from 25 to 48 °C. To calculate the strain, we took the length of a free‐standing LCE sample at 25 °C as the reference, so negative and positive strain represent material contraction and elongation, respectively. Figure S5 (Supporting Information) shows the repeatability of the stress–strain curves of the monodomain LCEs. The material maintains similar modulus and strength across this temperature range. Figure 4B illustrates the temperature‐dependent actuation stress of a monodomain LCE with a fixed length. When the length of a monodomain LCE is fixed, the actuation stress increases monotonically with the temperature and eventually results in a material failure at ≈1 .7MPa. This behavior aligns with the testing results of the stress–strain curves in Figure 4A. As shown in Figure 4C, the stress relaxation of a monodomain LCE at different temperatures indicates a stress decrease of <10% over 3 h. This duration is longer than the typical length of a compression massage session, which usually lasts between 30 min to an hour.^[^ ^31^ ^]^ Last, Figure 4D shows the durability of a monodomain LCE through 1000 times of loading and unloading cycles, simulating the repeated application and removal of the stocking. The observed stress degradation is <10%, which highlights the robustness of the material, affirming its suitability for dynamic compression therapy applications.

#### 2.3.2 Design and the Integration of a Stretchable Heating Element

**Figure 5:**
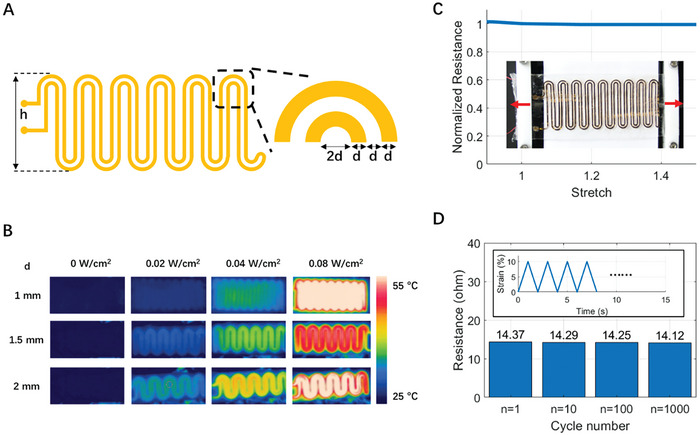
Thermomechanical characterizations of a stretchable heating element. A) Geometry and dimensions of the heating pattern. The number of serpentines is for illustration purposes. B) IR images of the heating pattern with line widths *d* from 1 mm to 2 mm with different heating power. C) The heating pattern with *d*  =  1 mm maintains its electrical resistance with a stretch between 0.9 and 1.4. The inserted picture shows the testing setup with a house‐made uniaxial stretcher to precisely control the stretch. D) The change of the electrical resistance of the heater as a function of the cycle number with the pattern width *d*  =  1 mm.

Another essential component of the LCE‐based dynamic stocking is its heating element, which was first fabricated from a thin copper film using a contour cutter to achieve the desired serpentine shape. We then sandwiched the heater between two monodomain LCE sheets, forming the actuating segment of the stocking. The serpentine design and dimensions of the heating pattern are shown in **Figure** **5**A, where we kept the *h*  =  40 mm and varied the line width *d* in this study. The number of serpentines in Figure 5A is only for illustration purposes and it may vary for different line width *d* and the overall dimension of the LCE‐based dynamic stocking.

Figure 5B illustrates the temperature evaluation of the heating pattern with the copper line widths *d* ranging from 1 to 2 mm, sandwiched between two monodomain LCE layers and fixed in length. We used an IR camera to assess the equilibrium temperature distribution across the patterns with *d*  =  1 mm, *d*  =  1.5 mm, and *d*  =  2 mm at varying input powers. The design with a 1 mm line width was identified as the heater due to its uniform temperature distribution between 25 and 50 °C. Therefore, we chose the heating pattern with *d*  =  1 mm for the study and the rest of the characterizations.

As depicted in Figure 5C, we assessed the mechanical durability of the heating element with *d =* 1 mm under compression and tension. In the tests, we sandwiched the heating pattern between 2 stretched Very High Bonding (VHB) sheets and used a custom uniaxial stretcher, shown in the insert, for precise manipulation of the assembly. The heating element demonstrated consistent electrical resistance through 10% compression to 50% tension, affirming its robustness as a heating component.

Durability testing of the heating element, shown in Figure 5D, involved 1000 loading cycles to 10% strain. We chose the loading rate of 10% s^−1^ to mimic the practical LCE‐based static stocking application. The electrical resistance of the heating element remained unchanged throughout the 1000 cycles, confirming its suitability for repeated use in the LCE‐based dynamic stocking.

#### 2.3.3 Performance of the LCE‐Based Dynamic Stocking

**Figure 6:**
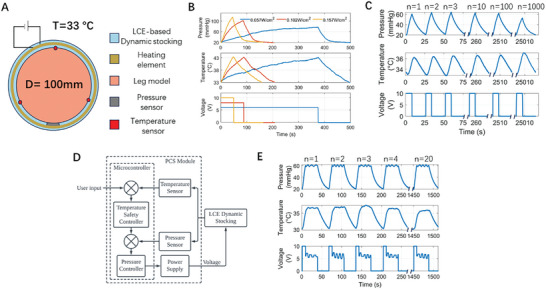
Characterization of the performance of an LCE‐based dynamic stocking. A) Schematics of the experimental setup. B) Characterization of interfacial pressure of LCE‐based dynamic stocking at 43 °C, with various DC power inputs. C) Under cyclic voltage input, the dynamic stocking generates intermittent compression cycles, where the interfacial pressure and temperature are measured as a function of time. The pressure cycles remain consistent for up to 1000 cycles. D) Block diagram of closed loop pressure feedback control for controlling the interfacial pressure profile of the LCE‐based dynamic stocking. E) With pressure feedback control, the interfacial pressure profile of the LCE‐based dynamic stocking can be programmed. The temperature and the applied voltage were measured as a function of time.

We constructed the LCE‐based dynamic stocking with a DC power module (V_p_) and a layered structure comprising two monodomain LCE sheets and one stretchable heater, with the LCE layers covalently bonded during the second step crosslink of the monodomain LCE synthesis. As shown in **Figure** **6**A, the experimental setup for the characterization of LCE‐based dynamic stocking consists of a rigid leg model of 100 mm diameter and, a pressure sensor and three thermistors. We conducted the characterization by securing both ends of the LCE‐based dynamic stocking to the leg model. To mimic the working environment of the dynamic compression treatment, prior to each characterization, we heated up the stocking to 33 °C, which is around human skin temperature. The detailed experimental setup can be found in Figure S6 (Supporting Information).

Figure 6B illustrates the capability of LCE‐based dynamic stocking to modulate interfacial pressure through joule heating with varying power inputs. We set the temperature threshold to 43 °C, which is approximately the maximum skin temperature humans can endure for up to 8 h without damage.^[^ ^32^ ^]^ As the power input increases from 0.057 W cm^−2^ to 0.157 W cm^−2^, the time needed to heat the LCE‐based dynamic stocking from 33 °C to 43 °C reduces from 375 s to 49 s, indicating that the compression cycle can be significantly shortened by increasing the power consumption. More importantly, the peak pressure increases from 76.9 mmHg to 113.7 mmHg, allowing the LCE‐based dynamic stocking to achieve higher pressure within a shorter time, which is a desired feature of the dynamic compression device. Therefore, 0.157 W cm^−2^ was selected as the input power for the LCE‐based dynamic stocking, balancing performance with energy conservation.

As illustrated in Figure 6C, we wrapped the LCE‐based dynamic stocking around the leg model to exert an initial interfacial pressure of 20 mmHg. Periodic power input was applied to the LCE‐based dynamic stocking, where *V_p_* =  10V, *t_on_* =  8s, and *t_off_* =  20s. Cyclic pressure profiles and temperature profiles were measured. With this power input, the heating and cooling cycle takes ≈30 s, similar to intermittent pneumatic devices.^[^ ^31^ ^]^ Noticeably, the peak pressure of 60 mmHg is consistently maintained across hundreds of cycles, demonstrating the good performance of the stocking for elastomer‐based smart compression therapy. The device temperature remained stable throughout these cycles, indicating safe operation within a comfortable range for human skin.

Figure 6E illustrates the controllability of the pressure profile generated by an LCE‐based dynamic stocking enabled by pressure feedback control (Figure 6D). In Figure 6E, we demonstrated that the dynamic stocking can maintain a constant pressure level at 60 mmHg for 30 s for each compression cycle. We used the interfacial pressure as a feedback signal to control the voltage input, therefore changing the applied temperature to regulate the pressure level close to the prescribed value. This capability suggests that more complicated pressure profiles could be realized with the measured thermomechanical properties of a monodomain LCE and the theoretical model in Equation (1), enhancing the system's adaptability for various therapeutic purposes.

**Figure 7:**
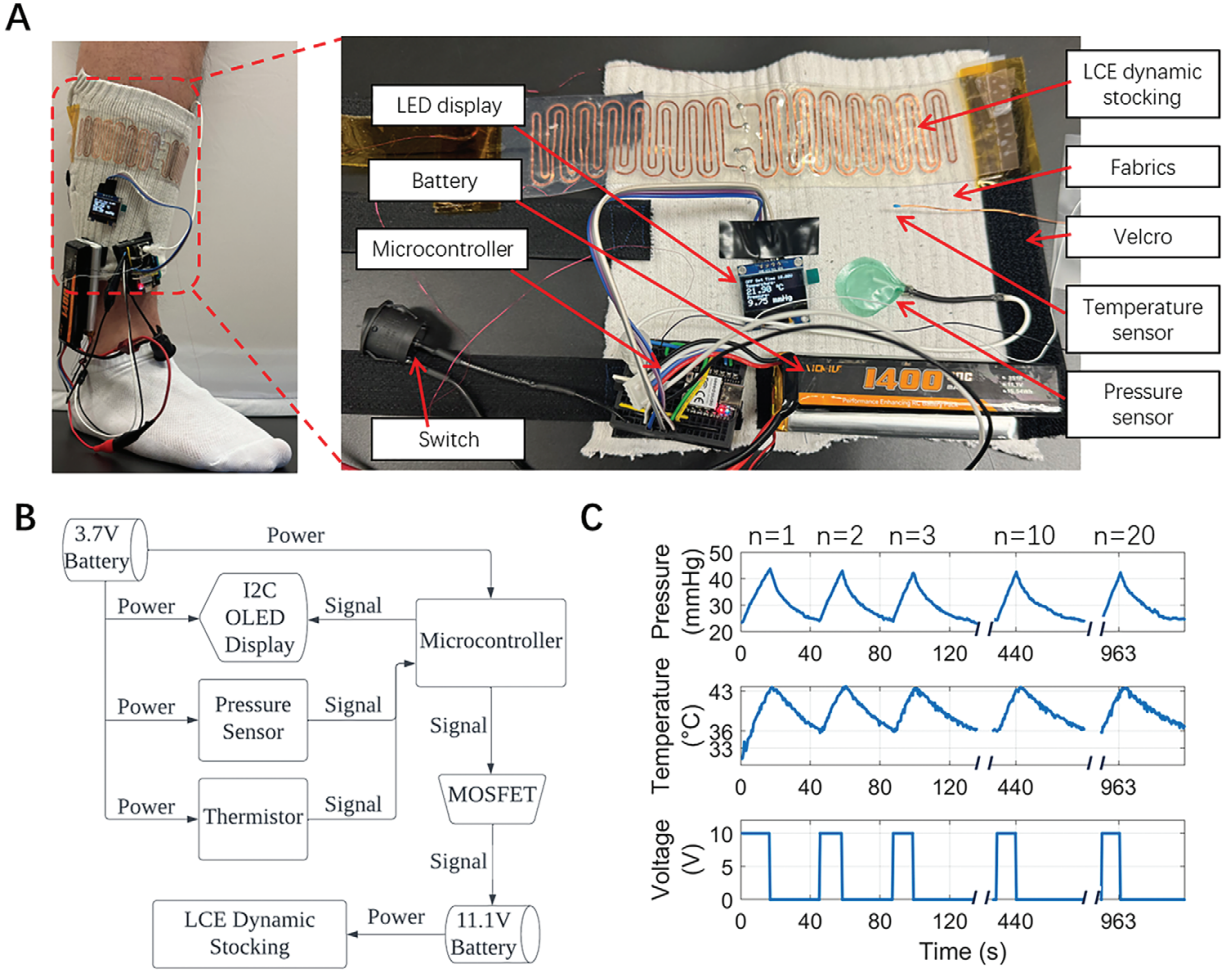
Application of an untethered and wearable LCE‐based dynamic compression device on a human leg. A) Design of the portable LCE compression device and its application on the human leg. The LCE‐based dynamic stocking and the PCS module are attached to a piece of fabric with Velcro straps. B) The working principle and electronic design of the PCS module. C) The performance of the untethered LCE‐based dynamic compression device on a human leg for 20 cycles, exerting interfacial pressure from 23 to 43 mmHg. The maximum working temperature was kept at 43 °C.

In addition, we developed an untethered and wearable LCE‐based dynamic compression device. As illustrated in **Figure** **7**A, the compression device is applied to a human calf. The LCE‐based dynamic stocking, PCE module, and Velcro straps are secured to a cotton fabric substrate, allowing easy application of the integrated device. This design not only allows patients to easily apply and adjust the device to fit various leg sizes by themselves but also incorporates a fabric layer as a thermal insulating layer, further reducing the risk of skin damage caused by heat. In contrast to conventional pneumatic compression devices that are typically stationary and cumbersome, the wearable LCE compression device only weighs 186 g including the batteries to support mobility, thereby not interfering with the daily activities of the patients.

Figure 7B details the working principle of the PCS module used for the dynamic compression device. A microcontroller is programmed to manage the power from an 11.1 V lithium polymer battery, activating the stocking on demand. The pressure sensor monitors real‐time interfacial pressure between the fabric and the leg, while the temperature sensor monitors the interface temperature between the LCE‐based dynamic stocking and the fabric. An LED display provides real‐time data on pressure, temperature, and voltage, enhancing user interaction. Figure S7 (Supporting Information) details the explanation of the circuit design.

Finally, we demonstrated the performance of a wearable LCE‐based dynamic compression device on a human leg. As shown in Figure 7C, by controlling the temperature between 36 to 43 °C, the compression device can exert an interfacial pressure from 23 to 43 mmHg on the leg. The microcontroller receives the temperature feedback signal from the temperature sensor and controls the applied voltage to the compression device. An IR video of the intermittent pressure cycles can be found in Movie S1 (Supporting Information). The peak interfacial pressure remained consistent for at least 20 cycles, indicating a consistent performance of the device. Each intermittent compression cycle was <50 s, which is comparable to pneumatic‐based compression devices that are typically ≈1 min.^[^ ^32^ ^]^ This timescale is much shorter than other thermally responsive compression devices, such as shape memory polymer‐based compression devices that take over half an hour for one cycle. With a 1400 mAh battery, the device can function up to 296 cycles that correspond to around 4‐h of compression sessions, where detailed calculations can be found in Text S2 (Supporting Information).

In the experiments shown in Figures 6 and 7, we used the same LCE‐based dynamic stocking on the rigid leg model and real human leg. We have noticed that although the temperature increase in the human leg demonstration is higher than in the stocking, a smaller interfacial pressure was generated. This is due to the higher compliance of the human leg. As the temperature increases, the diameter of the human leg decreases in response to the LCE actuation, leading to the shortening of the LCE. As a result, the actuation stress of LCE is smaller in human leg experiments. Such reduction of the interfacial stress can be easily compensated by slightly increasing LCE thickness.

## 3 Conclusion

Though various solutions have been proposed for compression therapy in the past, intrinsic limitations still exist. Both static and dynamic compression therapies often fail to maintain optimal pressure levels over the required duration, affecting therapeutic efficacy. In this study, we successfully addressed the challenges of both static compression and dynamic compression therapy by using the LCE as the primary material due to its unique thermomechanical properties.

For static compression stocking, we developed a polydomain LCE incorporated with PEGDA into the polymer network. The resulting polydomain LCE exhibits stress plateau over a large range of strain and negligible mechanical hysteresis, which can be harnessed to accommodate inconsistencies of stocking application, various limb sizes, and the interfacial pressure drop due to the deswelling of a leg. Moreover, the polydomain LCE shows negligible stress relaxation and good repeatability for over 1000 cycles of loading to 30% strain, which offers consistent interfacial pressure from the LCE‐based static stocking for a long duration.

For dynamic compression stocking, we introduced a monodomain LCE for the actuation, harnessing its reversible thermal actuation properties. The nematic‐isotropic phase transition of an LCE enables the LCE to exert sufficient pressure around the leg when it is heated slightly above human skin temperature. The embedded heater was carefully designed and characterized to ensure high temperature homogeneity and durability.

Our experiments have shown that an LCE‐based dynamic stocking can generate interfacial pressure from 20 to 60 mmHg with a temperature increase from 33 to 38 °C for over 1000 cycles. We further demonstrated that a controlled pressure profile can be achieved by using pressure feedback control. Moreover, the untethered and wearable LCE‐based compression device composed of a PCS module, fabric, and Velcro straps, successfully applied up to 43 mmHg of pressure to the human leg over 20 cycles. With a single battery charge, the device could support continuous intermittent pressure cycles for ≈4 h.

## 4 Experimental Section

### Materials

4‐(6‐(acryloyloxy)hexyloxy)phenyl‐4‐(6‐ (acryloyloxy)hexyloxy)benzoate (C6BAPE, Chemfish, 97%), 2,4,6‐Triallyloxy‐1,3,5‐triazine (TAC, Sigma‐Aldrich, 97%), Poly(ethylene glycol) diacrylate (PEGDA, Mn 500, Sigma‐Aldrich, 95%), 2,2′‐(ethylenedioxy) diethanethiol (EDDET, Sigma‐Aldrich, 95%), pentaerythritol tetrakis (3‐mercaptopropionate) (PETMP, Sigma‐Aldrich, 95%), dipropylamine (DPA, Sigma‐Aldrich, 98%), (2‐hydroxyethoxy)‐2‐methylpropiophenone (HHMP; Sigma‐Aldrich; 98%), and all the solvents were used as received without further purification. All the chemical structures can be found in Figure S8 (Supporting Information).

### Fabrication of an LCE‐Based Static Stocking

The polydomain LCE film is prepared via base‐catalyzed thiol‐acrylate Michael addition reaction followed by a second‐stage photopolymerization.^[^ ^33^ ^]^ First, C6BAPE (10.0000 g), PEGDA (0.7396), and TAC (0.3673 g) were dissolved in toluene (3.1000 g) at 80 °C for 30 min. Second, a mixture of chain extender EDDET (1.9018 g) and crosslinker PETMP (1.5293 g) was added to the solution. For the above chemicals, a stoichiometry was maintained of

(2) $$\begin{array}{cc} & 2n_{C6BAPE}:2n_{PEGDA}:3n_{TAC}:4n_{PETMP}:2n_{EDDET}\\ & \;=0.84:0.06:0.1:0.5:0.5 \end{array}$$

After the mixture above is fully dissolved, the photo‐initiator HHMP (0.0772 g) and catalyst DPA solution (1 wt%, 3.24 g) were sequentially added to the solution. Third, it was vigorously mixed and vacuumed the solution for 5 min to remove the air trapped in the solution. Then, the solution was poured into a compression‐assisted mold for 24 h in a dark environment and dried at 80 °C for another 24 h. Finally, the loosely crosslinked LCE was placed on a 0 °C cold plate, and the LCE was left under UV (365 nm) for 60 min to obtain the polydomain LCE. The LCE‐based static stocking was further fabricated by bonding a polydomain LCE and Velcro strips (hook and loop) with VHB tape, as shown in Figure 1A.

### Fabrication of an LCE‐Based Dynamic Stocking and Wearable Compression Device

First, a similar method of the polydomain LCE synthesis was adopted for the monodomain LCE film, where the chemical ratio was kept at:

(3) $$2n_{C6BAPE}:3n_{TAC}:4n_{PETMP}:2n_{EDDET}=0.9:0.1:0.5:0.5$$

After the loosely crosslinked LCE was obtained, the LCE was stretched and fixed it at λ  =  1.8 on a 0 °C cold plate to avoid LCE temperature increase. To fabricate the heating pattern, a contour cutter (Maker 3, Cricut, USA) equipped was used with a fine point blade (30‐degree blade angel) to cut a copper–polyimide bilayer sheet (Bate Electronics, China) on an adhesive mat (Cricut, USA). The copper–polyimide bilayer sheet consists of a copper layer of 2 microns and a polyimide layer of 25 microns. Then, two 30 cm‐long copper wires (36AWG, BNTECHGO, China) were soldered to the heating element and transferred the heating element using water‐soluble tape (AQUASOL, USA) to the pre‐stretched LCE sheet. After gently washing off the water‐soluble tape and drying the water at room temperature, another layer of pre‐stretched LCE sheet was carefully put on top of the first layer. Finally, the LCE‐heater‐LCE sandwich structure was left under UV (365 nm) for 60 min to obtain the LCE‐heater‐LCE sandwich structure as the LCE‐based dynamic stocking.

A sewing machine (3160QOV, Janome, Japan) is used to stitch the Velcro (hook and loop) straps onto a piece of cotton fabric as the substrate material for the wearable LCE‐based dynamic compression device. As shown in Figure 7A, the LCE‐based dynamic stocking, OLED display, and PCS module were attached to the cotton fabrics in different positions with Velcro on the back.

### Design of PCS (Power, Control, Sensor) Module

The PCS module consists of a lithium‐polymer battery (11.1 V, 1400 mAh, Vicmile, China) for powering the heating element, another lithium‐polymer battery (3.7 V, 3700 mAh, EEMB, China) for powering the electronics, a MOSFET and a microcontroller (Xiao PR2040, DigiKey, USA) for controlling voltage, 1 negative temperature coefficient thermistors (10kOhm, Murata Electronics, USA) for temperature sensing, a pressure sensor (5PSI, Honeywell, USA) and a homemade sensing pouch (1mm thick) for pressure monitoring, and an I2C OLED display (0.96″, HiLetgo, China) for real‐time data visualization. The PCS module first controlled the power supply using the microcontroller with a programmed input signal. Then, the microcontroller continuously monitors the analog input from the thermistors and the pressure sensor. Finally, the microcontroller processes the sensor data and sends it to the computer for data recording and to the OLED for display. The detailed circuit design of the PCS module can be found in Figure S7 (Supporting Information).

The pressure sensing module consists of a pressure sensor (5PSI, Honeywell, USA) and a homemade sensing pouch. The sensing pouch was made by hot pressing (MODEL 4386, Carver) 2 layers of thermoplastic polyurethane (TPU) with an isolating polyethylene terephthalate (Mylar) layer. The pouch was connected to the pressure sensor using silicone tubing and heat shrink to ensure airtightness. Detailed dimensional design and experimental pictures can be found in Figure S9 (Supporting Information).

### Characterization

The uniaxial tensile and cyclic loading tests were performed using a tensile tester (5965, Instron) with a 10N static load cell. The 1% s^−1^ strain rate was used for this study.

The uniaxial tensile tests above room temperature and stress relaxation tests were performed using a dynamic mechanical analyzer (RSA‐G2, TA Instrument) at specified temperatures. A 1% s^−1^ strain rate was adopted for the tensile tests.

The IR images and videos were captured by the IR camera (C3‐X, FLIR), where the temperature reading was calibrated to match the actual temperature measured by a separate thermocouple.

The electrical resistance of the heating element was measured by applying a 5 V DC voltage to the heating pattern and recording the current value. The electrical resistance was then calculated accordingly using Ohm's law:

(4) $$R=\frac{U}{I}$$

where *U* is the applied voltage, and *I* is the current.

Preliminary cost analyses were performed to address the potential concerns of material cost for both LCE‐based static stocking and LCE‐based dynamic stocking in Tables S3 and S5 (Supporting Information), respectively, and further compared with their corresponding commercially available products in Tables S4 and S6 (Supporting Information). It was anticipated that scaling up production would help reduce material costs.

## Conflict of Interest

The authors declare no conflict of interest.

## Author Contributions

G.D. and S.C. designed research; G.D., F.Z., and Z.G. performed research; G.D. analyzed data; G.D. and S.C. wrote the paper.

## Supporting information



Supporting Information

Supplemental Movie 1

## Data Availability

The data that support the findings of this study are available from the corresponding author upon reasonable request.
