# Supplementary material for: Liquid Crystal Elastomer for Compression Therapy
Source: Adv Healthc Mater. 2024 Dec 4;14(3):2402881. doi: 10.1002/adhm.202402881 (PMC11773120; doi:10.1002/adhm.202402881)
Supplement: Supplementary file 1 — Supporting Information [file ADHM-14-0-s002.docx]

Supporting Information

Liquid Crystal Elastomer for Compression Therapy

Gaoweiang Dong, Fangchen Zhao, Zongyu Gao, Shengqiang Cai*

**This file includes:**

Supplementary Text S1-S2

Table S1 to S6

Figure S1 to S9

**Other Supplementary Information for this manuscript includes the following:**

Movies S1

Supplementary Text

**Text S1**

The chemical composition of the following samples are:

LCE_0PEGDA: ${2n}_{C6BAPE}:{2n}_{\mathrm{PEGDA}}:{3n}_{\mathrm{TAC}}:{4n}_{\mathrm{PETMP}}:{2n}_{\mathrm{EDDET}}=0.90:0.00:0.1:0.5:0.5$ (1)

LCE_4PEGDA: ${2n}_{C6BAPE}:{2n}_{\mathrm{PEGDA}}:{3n}_{\mathrm{TAC}}:{4n}_{\mathrm{PETMP}}:{2n}_{\mathrm{EDDET}}=0.86:0.00:0.1:0.5:0.5$ (2)

LCE_6PEGDA: ${2n}_{C6BAPE}:{2n}_{\mathrm{PEGDA}}:{3n}_{\mathrm{TAC}}:{4n}_{\mathrm{PETMP}}:{2n}_{\mathrm{EDDET}}=0.84:0.06:0.1:0.5:0.5$ (3)

LCE_8PEGDA: ${2n}_{C6BAPE}:{2n}_{\mathrm{PEGDA}}:{3n}_{\mathrm{TAC}}:{4n}_{\mathrm{PETMP}}:{2n}_{\mathrm{EDDET}}=0.82:0.08:0.1:0.5:0.5$ (4)

LCE_10PEGDA: ${2n}_{C6BAPE}:{2n}_{\mathrm{PEGDA}}:{3n}_{\mathrm{TAC}}:{4n}_{\mathrm{PETMP}}:{2n}_{\mathrm{EDDET}}=0.80:0.10:0.1:0.5:0.5$ (5)

**Text S2**

The electrical resistance $R$ of the heating element is 11.5 ohms. The battery used to power the heating elements has a capacity of $It=1400mAh$ at $U=11.1V$. By using Ohm’s law:

$I=\frac{U}{R}$ . (6)

We calculated that the battery could continuously power the heating element for around $t=1.45h$. For each compression cycle of the untethered and wearable LCE-base compression device, the average heating time and cooling time are $t_{heating}=17s$ and $t_{cooling}=28s$, respectively. Therefore, the total running time $t_{total}=3.84h$ for the compression device can be calculated as follows:

$t_{total}=t\times\frac{t_{heating}{+t}_{cooling}}{t_{heating}}$ . (7)

**Table S1**: Static compression therapy devices

| Type | Design | Pros/Cons |
| --- | --- | --- |
| Inelastic bandage^[2–4]^ | Non-stretchable bandages wrapped around the limb. | Pros: customizable pressure; low cost  Cons: high dependence of application skill; pressure loss over time |
| Elastic stocking^[5,6]^ | Stretchable hosiery-type garments in long sock shape. | Pros: prescribed pressure range; easy application  Cons: pressure depending on limb size; pressure drop during leg deswelling |
| **LCE-based static stocking (current work)** | **Polydomain LCE incorporated with Velcro and fabrics.** | **Pros: consistent pressure over a wide range of leg sizes; good tolerance on application errors; negligible pressure drop during leg deswelling** |

**Table S2**: Dynamic compression therapy devices

| Type | Design | Pros/Cons |
| --- | --- | --- |
| Pneumatic stocking^[7]^ | Pneumatic pumps inflate soft air bladders in a boot-like structure. | Pros: precise and controllable pressure up to 400 mmHg  Cons: bulky; noisy; not portable |
| Motor-driven stocking^[8,9]^ | Motors drive tension wires around a rigid stocking. | Pros: rapid actuation speed; high pressure up to 87.3 mmHg  Cons: hard to integrate into garments; rigid component |
| Shape memory alloy-based stocking^[10]^ | SMA wires are integrated into fabrics to create compression. | Pros: seamless integration with textile  Cons: high operational temperatures; rigid component |
| Dielectric elastomer-based stocking^[11]^ | The soft actuator consists of silicone rubber and electrodes and integrates with Velcro. | Pros: precise and rapid pressure control up to 52.1 mmHg  Cons: high voltage; low pressure range from 14.4 to 21.5 mmHg |
| Shape memory polymer-based stocking^[12–16]^ | Shape memory polymers are integrated into textiles with a heating layer. | Pros: High actuation stress generates pressure up to 45 mmHg  Cons: stress relaxation; stiff glassy state at room temperature |
| **LCE-based dynamic stocking**  **(current work)** | **Monodomain LCE incorporates with heating element and PCS module.** | **Pros: controllable pressure profiles up to 113.7 mmHg; compression cycle less than 60s; negligible pressure decay over 1000 cycles** |

**Table S3**: Material cost estimation of LCE-based static stocking

| Name | Unit | Price | Source |
| --- | --- | --- | --- |
| C6BAPE | 20 g | $50 | Chemfish, Japan |
| PEGDA | 1.48 g | $0.36 | Sigma-Aldrich, USA |
| TAC | 0.72 g | $0.52 | Sigma-Aldrich, USA |
| EDDET | 3.80 g | $1.11 | Sigma-Aldrich, USA |
| PETMP | 3.06 g | $0.44 | Sigma-Aldrich, USA |
| DPA | 0.06 g | $0.02 | Sigma-Aldrich, USA |
| HHMP | 0.16 g | $0.75 | Sigma-Aldrich, USA |
| Toluene | 13 g | $0.62 | Sigma-Aldrich, USA |
| **Sum** |  | **$53.82** |  |

**Table S4**: Cost comparison of LCE-based static stocking and other commercial compression stockings

| Brand | Unit price | Data Source  Oct. 15^th^ 2024 |
| --- | --- | --- |
| **LCE-based static stocking**  **(Current work)** | **$53.82** | **Estimation by chemical cost** |
| Amazon^®^ Basic Care | $23 | https://a.co/d/6j4cXOK |
| Terraform Sheer^®^ | $15 | https://a.co/d/2YYuSt2 |
| Laite Hebe^®^ | $15 | https://a.co/d/2hSQg6b |

**Table S5**: Material cost estimation of LCE-based dynamic stocking

| Name | Unit | Price | Source |
| --- | --- | --- | --- |
| C6BAPE | 21.4 g | $53.5 | Chemfish, Japan |
| TAC | 0.72 g | $0.52 | Sigma-Aldrich, USA |
| EDDET | 3.80 g | $1.11 | Sigma-Aldrich, USA |
| PETMP | 3.06 g | $0.44 | Sigma-Aldrich, USA |
| DPA | 0.06 g | $0.02 | Sigma-Aldrich, USA |
| HHMP | 0.16 g | $0.75 | Sigma-Aldrich, USA |
| Toluene | 13 g | $0.62 | Sigma-Aldrich, USA |
| copper–polyimide bilayer | 0.008 m^2^ | $1.02 | Bate Electronics, China |
| Lipo Battery  (11.1 V, 1400 mAh) | 1 | $20 | Vicmile, China |
| Lipo Battery  (3.7 V, 3700 mAh) | 1 | $15.5 | EEMB, China |
| Microcontroller  (Xiao PR2040) | 1 | $10 | DigiKey, USA |
| I2C OLED display  (0.96’’) | 1 | $2 | HiLetgo, China |
| Velcro  (hook and loop) | 0.004 m^2^ | $0.8 | QTBLY Direct, USA |
| **Sum** |  | **$106.64** |  |

**Table S6**: Cost comparison of LCE-based dynamic stocking and other commercial pneumatic dynamic stocking

| Brand | Unit price | Data Source  Oct. 15^th^ 2024 |
| --- | --- | --- |
| **LCE-based dynamic stocking**  **(Current work)** | **$106.64** | **Estimation by component cost** |
| Cincom^®^ | $490 | https://a.co/d/d8nect2 |
| QUINEAR^®^ | $360 | https://a.co/d/aIXxMq3 |
| Compex^®^ | $500 | https://a.co/d/6j4cXOK |


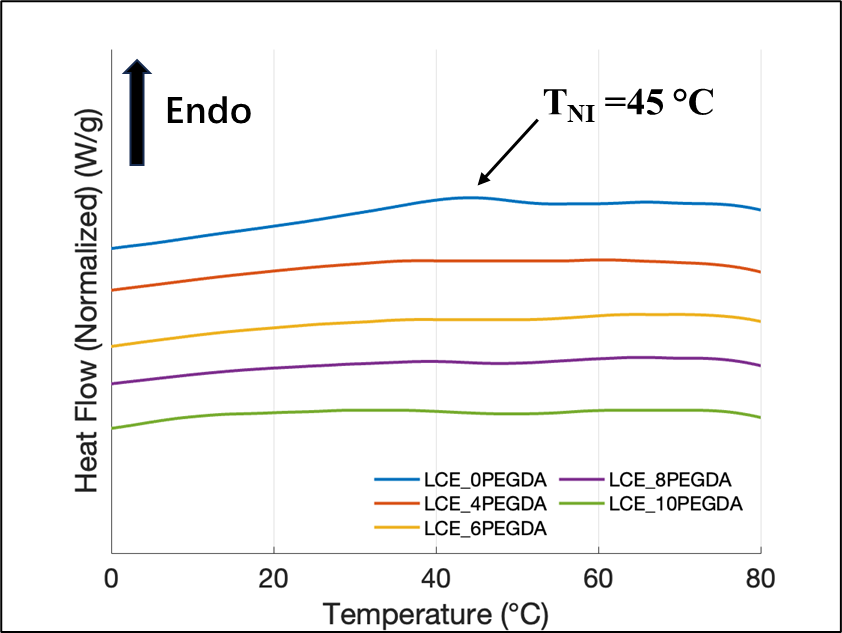


**Figure S1.** Differential Scanning Calorimetry (DSC) of polydomain LCEs with various PEGDA content.


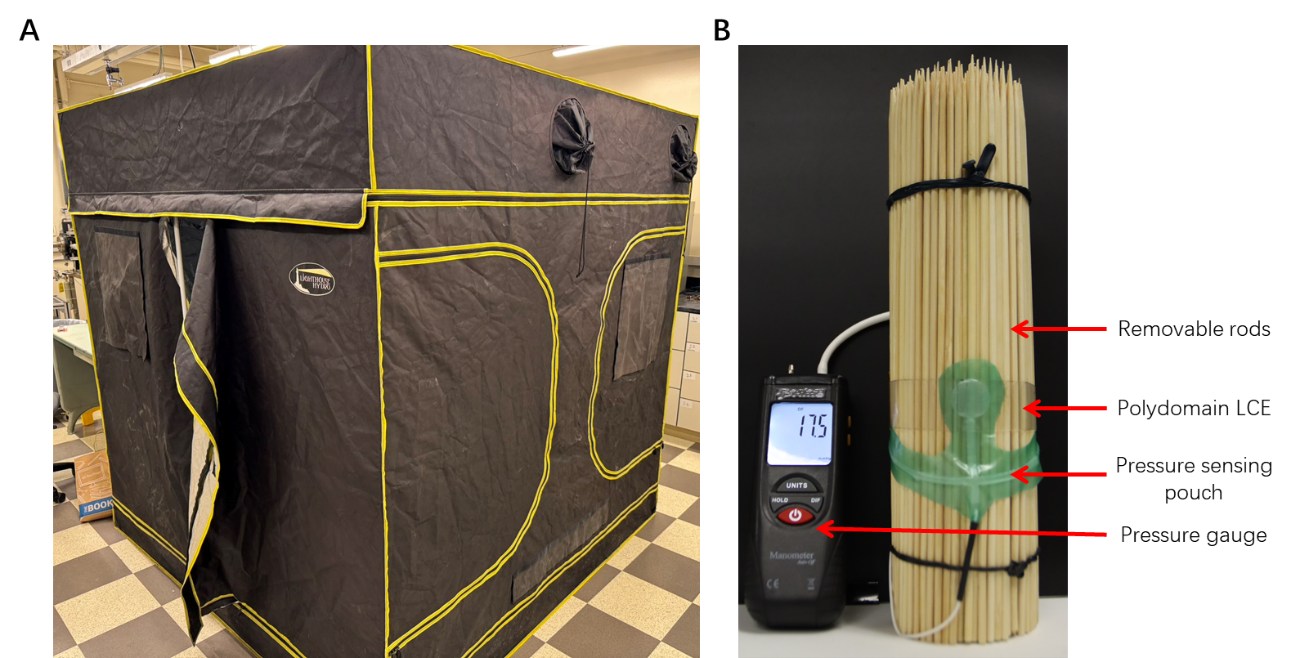


**Figure S2.** Experimental setup of the static LCE stocking characterization (A) the homemade tent with controllable temperature to mimic the human skin temperature, which is around 33 °C. (B) A bundle of removable rods is used to represent different sizes of legs. A differential pressure gauge and a homemade air pouch were placed between static LCE stockings and the bundle to measure the interfacial pressure.


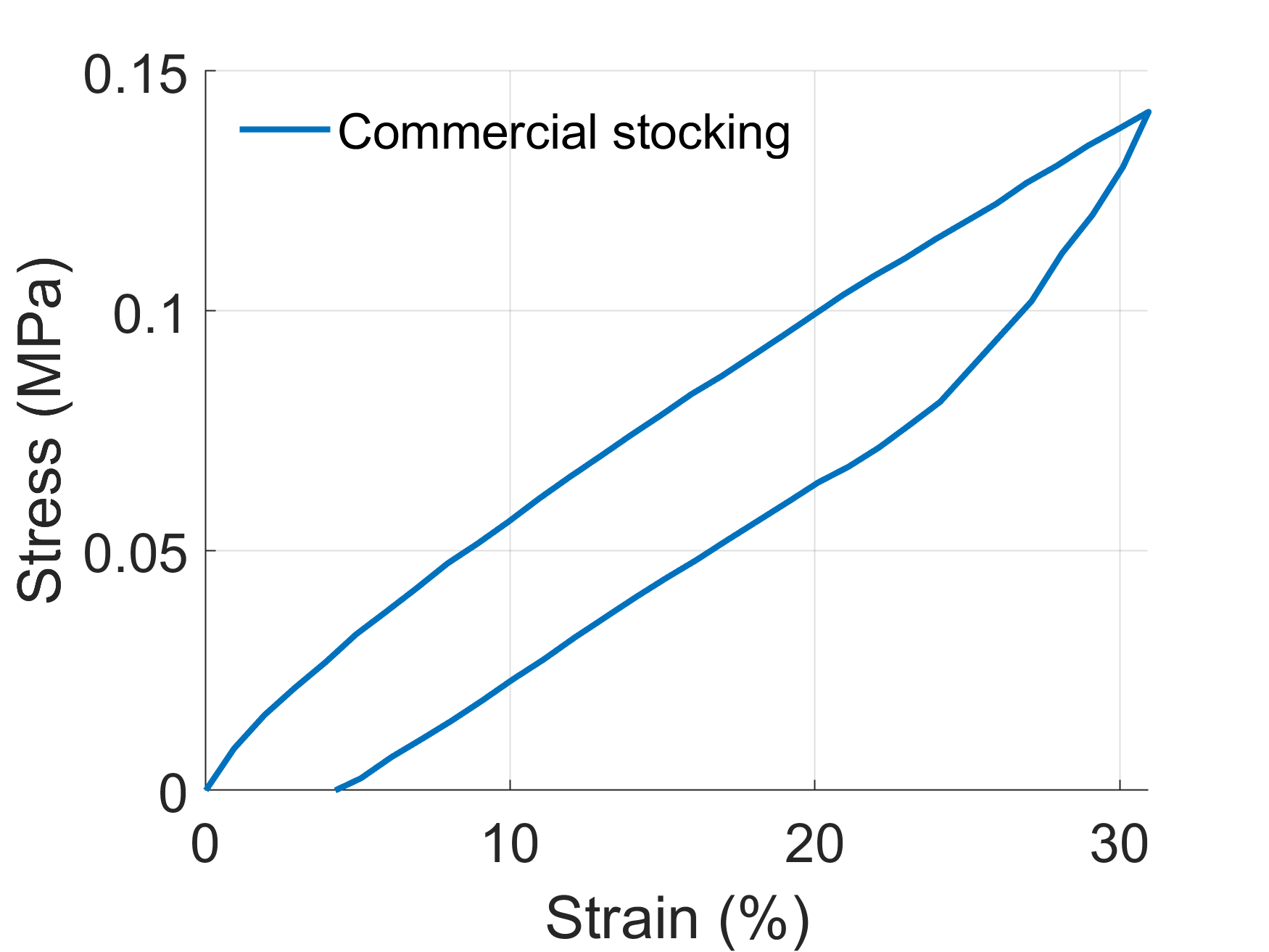


**Figure S3.** Tensile test of commercial elastic stocking (Amazon Basic Care, medium size) on the calf area along the course direction. The strain rate is 1% s^-1^.


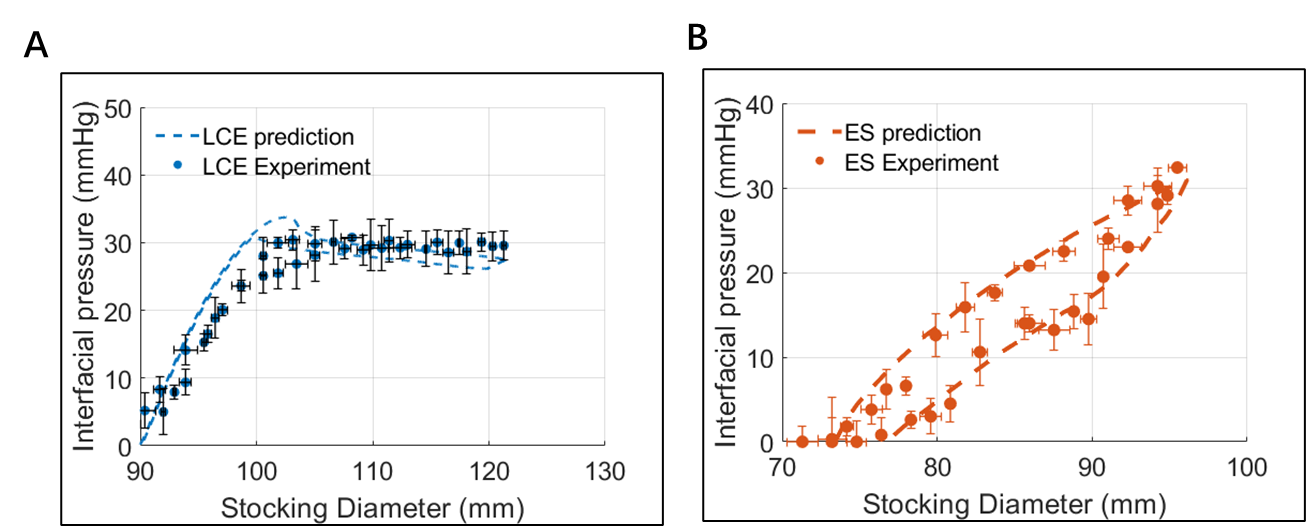


**Figure S4.** Prediction and measurement of interfacial pressure drop during unloading of (A) Figure 3C LCE static stocking and (B) Figure 3D commercial elastic stocking with error bar.


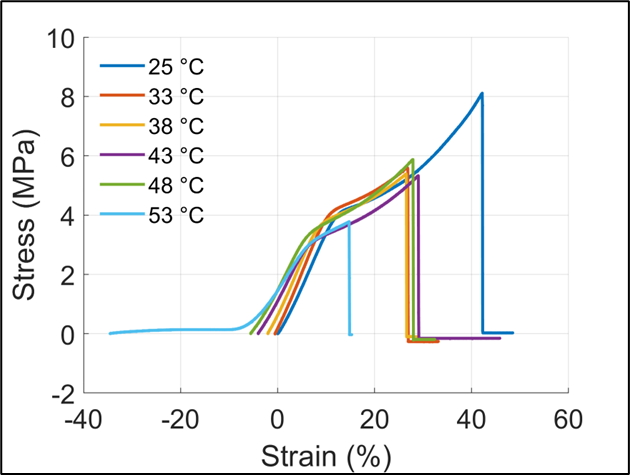


**Figure S5.** Additional characterization stress-strain relationships of monodomain LCE at various temperatures. The freestanding length under 25 °C was used as the reference to calculate the strain.


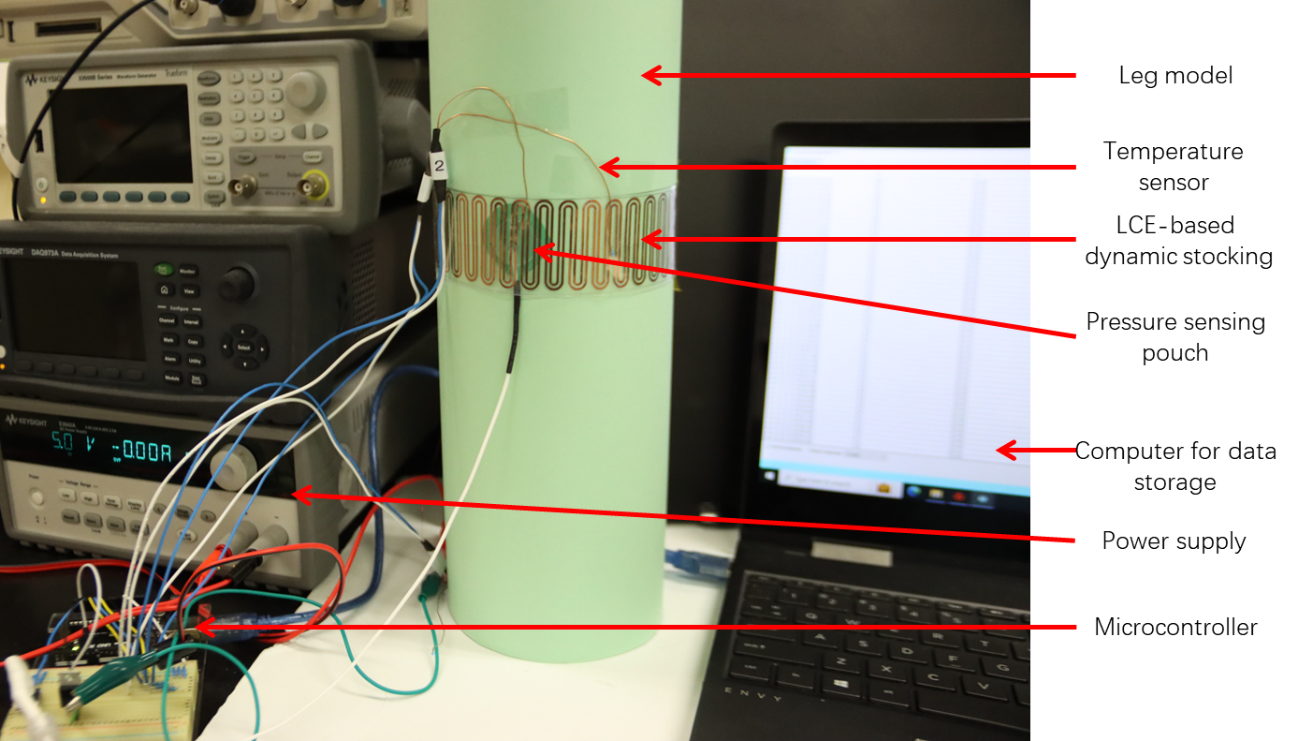


**Figure S6.** Experimental setup of the dynamic LCE stocking characterization


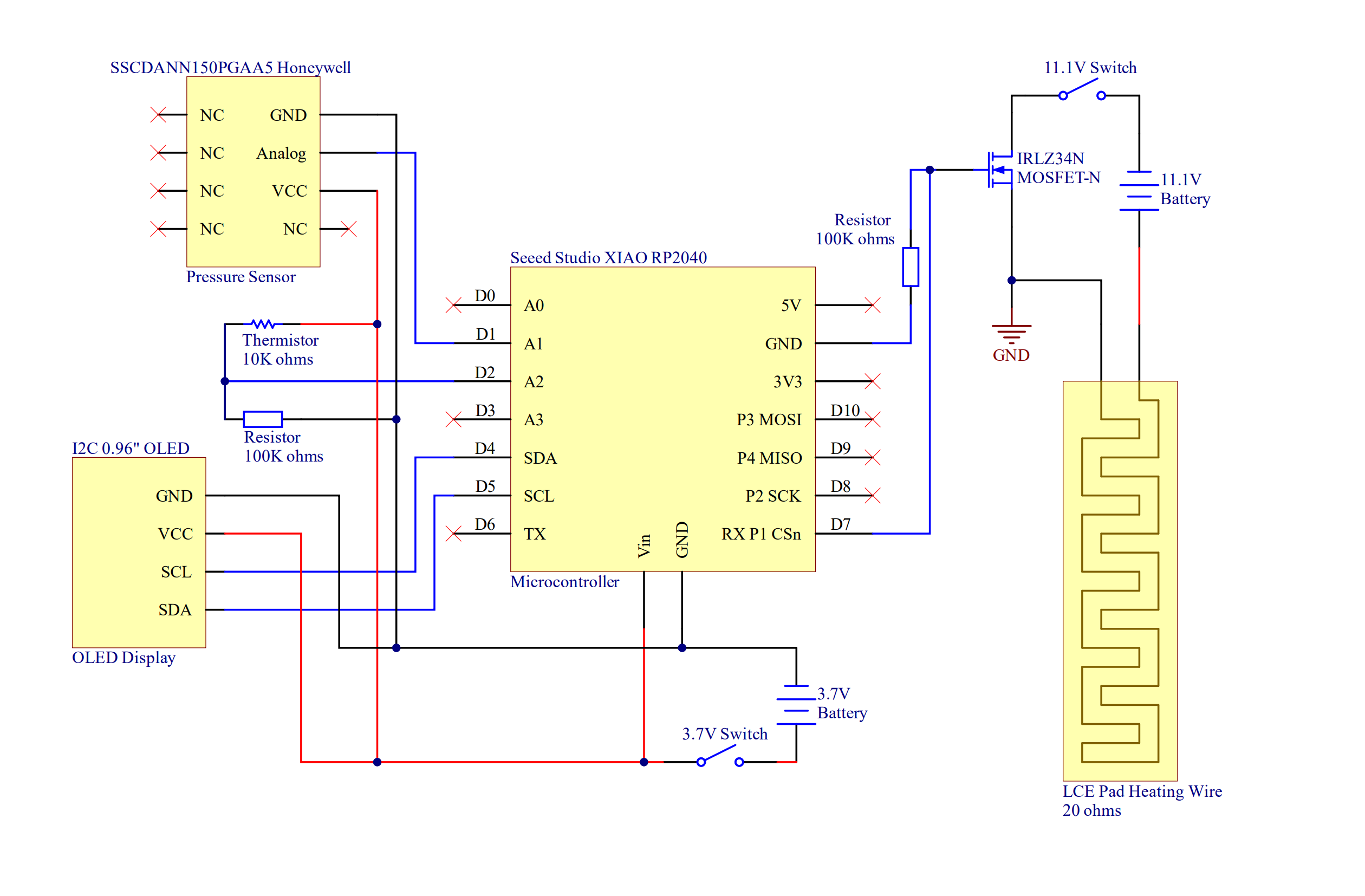


**Figure S7.** Circuit design of the PCS module

Overview:

The figure above shows the circuit design of the Power, Control, Sensor (PCS) module for the LCE-based dynamic stocking. The Seeed Xiao RP2040 microcontroller is responsible for continuously monitoring and processing data from the connected sensors. The microcontroller dynamically adjusts the control of the heating element and displays the temperature and pressure readings on the display. The temperature of the heating element is measured using a negative temperature coefficient (NTC) thermistor (10k ohms), whose resistance decreases with increasing temperature. The pressure between the stocking and the skin is measured using a piezoresistive silicon pressure sensor(SSCDANN150PGAA5, Honeywell), which outputs an analog voltage proportional to the pressure inside a sealed pouch. The heating element of the stocking is controlled via an N-channel metal-oxide-semiconductor field-effect transistor (MOSFET) (IRLZ34N), which acts as a switch to manage power delivery from the 11.1V Lithium Polymer battery based on commands from the microcontroller. A 0.96-inch I2C organic light-emitting diode (OLED) display is used for presenting real-time temperature, pressure, and voltage readings. A 3.7V Lithium Polymer battery powers the microcontroller, sensors, and display, while a separate 11.1V Lithium Polymer battery supplies power to the heating element. Each power system is equipped with its physical switch for user operation.

Circuit Design:

Thermistor Connection:

The 10k ohms thermistor is connected in a voltage divider configuration with a 100k ohms resistor. The junction between the thermistor and the fixed resistor is connected to an analog input pin (A2) on the Seeed Xiao RP2040. A 3.7V one-cell Lithium Polymer battery powers the thermistor.

Pressure Sensor Connection:

The pressure sensor's analog output is connected to an analog input pin (A1) on the Seeed Xiao RP2040. A 3.7V one-cell Lithium Polymer battery powers the pressure sensor.

MOSFET Control:

The gate of the N-channel MOSFET is connected to a digital output pin (D7) on the Seeed Xiao RP2040. The source is connected to the ground, and the drain is connected to the negative terminal of the 11.1V three-cell Lithium Polymer battery. The positive terminal of the 11.1V three-cell Lithium Polymer battery is connected to the heating wire.

I2C OLED Display Connection:

The SDA (data) and SCL (clock) pins of the OLED display are connected to the corresponding I2C pins (SDA and SCL) on the Seeed Xiao RP2040. A 3.7V one-cell Lithium Polymer battery powers the I2C OLED display.

Heating Element Connection:

The heating element connection in this system is designed to interface with an 11.1V three-cell Lithium Polymer battery. The MOSFET is used as a switching device to control the power flow to the heating element. Additionally, a power switch is incorporated to offer user control, allowing for the safe activation and deactivation of the heating element.

Calibration:

To ensure accurate and reliable measurements in experimental tests, it is essential to perform a linear calibration of both the pressure and temperature sensors using a pressure gauge (LEX1, Keller, Switzerland) and a k-type thermocouple thermometer prior to each test.

For the pressure sensor: Apply a series of known pressure values between 0-80 mmHg to the sensor by connecting it to a pressure meter using a push-in T-connector. These values should cover the entire range of the sensor and be evenly spaced.

For the temperature sensor: Expose the sensor to a series of known temperature points between 0-100°C using a thermocouple thermometer. These points should be evenly distributed across the operating range.

Plot the recorded sensor outputs versus the known values of pressure or temperature. Perform a linear regression analysis to determine the best-fit line through the data points. This line represents the calibration curve for the sensor. From the linear regression, determine the slope and intercept of the calibration curve. These constants will be used to convert the sensor’s raw data into meaningful units during the tests. To verify the accuracy of the calibration, apply a few known values again and compare the sensor readings with the expected results based on the calibration curve. Adjustments may be required if there are significant discrepancies.

Safety and Considerations:

The compression device is equipped with critical safety features, including a temperature safety setting of 46 °C to prevent overheating and a pressure safety setting of 80 mmHg to avoid excessive compression, ensuring both the efficacy and safety of the device during operation. We used a suitable MOSFET that can handle the voltage and current requirements of the external load.


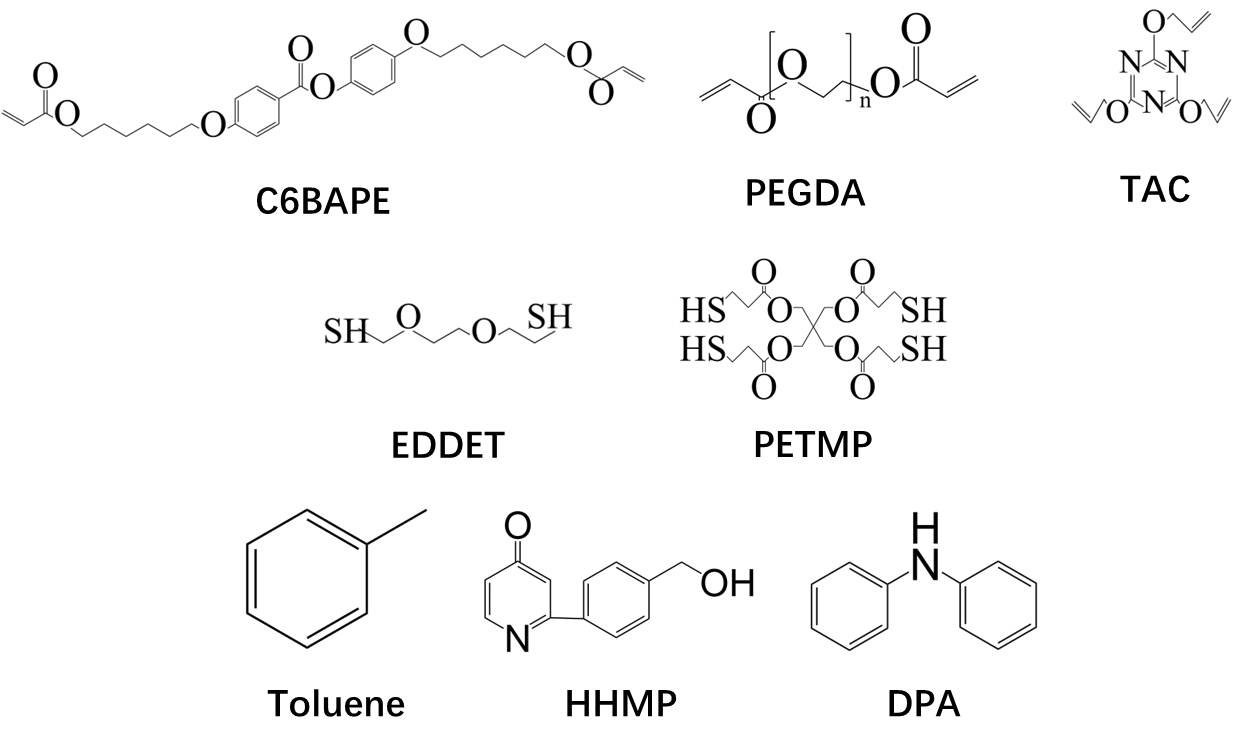


**Figure S8.** Chemical structures of 4-(6-(acryloyloxy)hexyloxy)phenyl-4-(6- (acryloyloxy)hexyloxy)benzoate (C6BAPE), 2,4,6-Triallyloxy-1,3,5-triazine (TAC), Poly(ethylene glycol) diacrylate (PEGDA, Mn 500), 2,2'-(ethylenedioxy) diethanethiol (EDDET), pentaerythritol tetrakis (3-mercaptopropionate) (PETMP), Toluene, (2-hydroxyethoxy)-2-methylpropiophenone (HHMP), and dipropylamine (DPA)


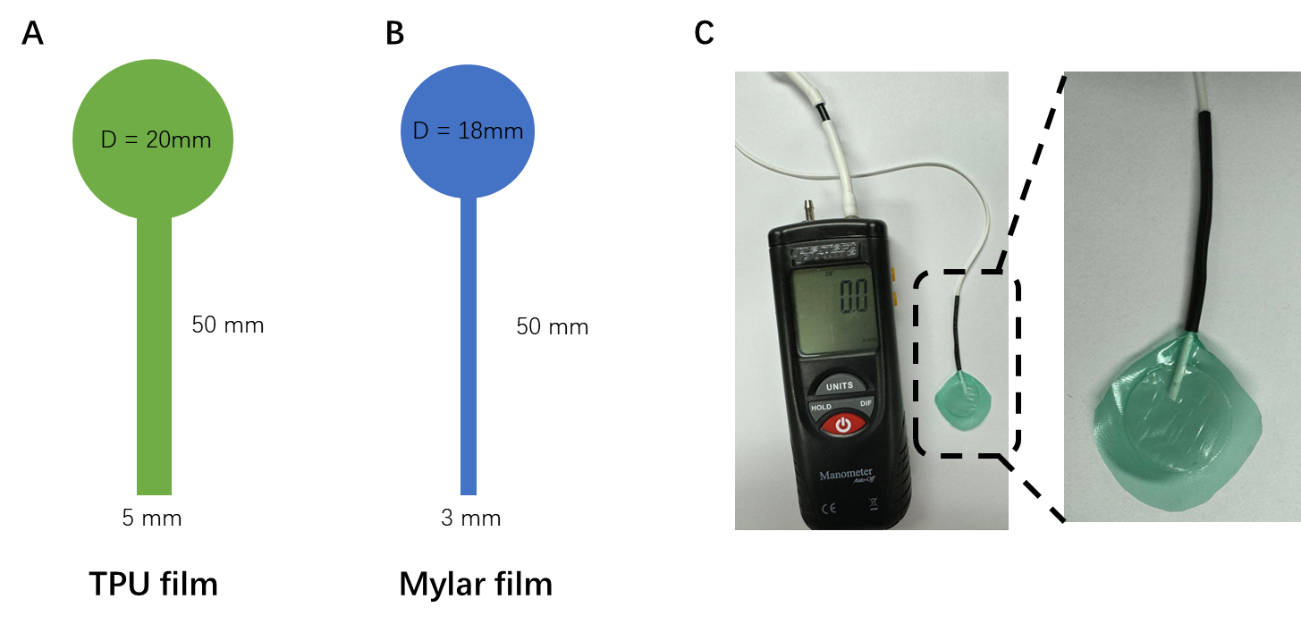


**Figure S9.** Dimension of the (A) TPU and (B) Mylar film for the pressure sensing pouch. C) Experimental picture of the pressure sensing pouch.

**Movie S1.** IR video of an untethered and wearable LCE-based dynamic compression device on a human leg.
